# Linkage Analysis and QTL Mapping Using SNP Dosage Data in a Tetraploid Potato Mapping Population

**DOI:** 10.1371/journal.pone.0063939

**Published:** 2013-05-21

**Authors:** Christine A. Hackett, Karen McLean, Glenn J. Bryan

**Affiliations:** 1 Biomathematics and Statistics Scotland, Invergowrie, Dundee, United Kingdom; 2 The James Hutton Institute, Invergowrie, Dundee, United Kingdom; Kansas State University, United States of America

## Abstract

New sequencing and genotyping technologies have enabled researchers to generate high density SNP genotype data for mapping populations. In polyploid species, SNP data usually contain a new type of information, the allele dosage, which is not used by current methodologies for linkage analysis and QTL mapping. Here we extend existing methodology to use dosage data on SNPs in an autotetraploid mapping population. The SNP dosages are inferred from allele intensity ratios using normal mixture models. The steps of the linkage analysis (testing for distorted segregation, clustering SNPs, calculation of recombination fractions and LOD scores, ordering of SNPs and inference of parental phase) are extended to use the dosage information. For QTL analysis, the probability of each possible offspring genotype is inferred at a grid of locations along the chromosome from the ordered parental genotypes and phases and the offspring dosages. A normal mixture model is then used to relate trait values to the offspring genotypes and to identify the most likely locations for QTLs. These methods are applied to analyse a tetraploid potato mapping population of parents and 190 offspring, genotyped using an Infinium 8300 Potato SNP Array. Linkage maps for each of the 12 chromosomes are constructed. The allele intensity ratios are mapped as quantitative traits to check that their position and phase agrees with that of the corresponding SNP. This analysis confirms most SNP positions, and eliminates some problem SNPs to give high-density maps for each chromosome, with between 74 and 152 SNPs mapped and between 100 and 300 further SNPs allocated to approximate bins. Low numbers of double reduction products were detected. Overall 3839 of the 5378 polymorphic SNPs can be assigned putative genetic locations. This methodology can be applied to construct high-density linkage maps in any autotetraploid species, and could also be extended to higher autopolyploids.

## Introduction

Linkage analysis and QTL mapping studies are now widely used in diploid plant species and are becoming increasingly common in polyploid species. Recent linkage maps for autotetraploid species include maps for potato [1], [2], alfalfa [3]–[5], forage grasses *Dactylis glomerata* [6] and *Paspalum notatum* Flügge [7] and rose [8]. These maps typically consist of hundreds of markers, combining dominant markers, such as Amplified Fragment Length Polymorphisms (AFLP), with codominant markers such as Simple Sequence Repeats (SSR). For example, the potato map used in [1] has 453 mapped markers, while that of [2] has 242 mapped markers for one parent and 219 for the other. Dominant markers are not very informative about repulsion linkages between homologous chromosomes, while the more informative codominant markers tend to be scored in smaller quantities due to their inherently lower multiplex ratio. QTLs for many important traits have been identified using these maps, but higher-density maps would benefit these studies enormously.

New genotyping technologies, for example the Illumina Infinium platform employed here, as well as sequence based methods, such as RAD sequencing, or genotyping by sequencing (GBS) allow the generation of high density single nucleotide polymorphism (SNP) genotype data on mapping populations and other similar populations [9], [10]. Data from genotyping platforms, including Infinium data, are generally provided as allele intensities, and intensity ratios can be analysed to infer the allele dosage. Different statistical approaches for the dosage estimation have recently been proposed by [11], using a mixture of normal distributions, and [12], using a graphical Bayesian model. Current methodology, such as [13], for estimating linkage maps in polyploid species has, however, been developed for markers scored as presence/absence and does not make use of all the information available from the SNP dosages.

In this paper we extend the method of [13] for linkage map estimation in an autotetraploid population consisting of two parents and their F_1_ offspring to use SNP dosage information generated using Illumina Infinium technology. A mixture of normal distributions is used to infer SNP dosage from the intensity data. Our approach differs from that of [11] by using the expected genotype frequencies for each configuration. The steps of linkage map estimation – segregation analysis, marker clustering, calculation of pairwise recombination fractions and LOD scores, marker ordering and identification of parental phase – are modified to use dosage data. Finally, the method of [14] for QTL interval mapping in autotetraploids is developed to use SNP dosage data, and SNP intensity scores are mapped as QTL as a check on the estimated linkage maps.

These methods are applied to a full-sib potato population derived from a cross between processing clone 12610ab1 and the cultivar Stirling. Previous maps of this population using AFLP and SSR data have been published by [1], [15]–[17]. The parents and 190 offspring from this cross have been genotyped using an Infinium 8300 Potato SNP Array [18], and we have used the data obtained to estimate a high-density SNP map. The methods described here are applicable to the construction of high-density linkage maps in any autotetraploid species, and could potentially be extended to higher autopolyploids.

## Materials and Methods

### Plant Material

The population studied was the progeny of a cross between the breeding clone 12601ab1 and the cultivar Stirling. This tetraploid population has been extensively studied [1], [15]–[17], [19]–[21]. DNA from 190 individuals plus parents was extracted from frozen plant leaf tissue using the DNeasy Plant DNA Extraction kit (Qiagen Cat.No. 69106). The DNA was quantified using the Quant-iT™ PicoGreen® dsDNA Assay Kit and adjusted to a concentration of 30 ng/µl in dH2O and arrayed in microtitre plates for genotyping.

### SNP genotyping

SNP genotyping was carried out on the parental clones (Stirling, 12601ab1), as well as 190 offspring using the Infinium 8303 potato SNP array [18]. The SNPs in this array were selected from a set of biallelic, high confidence SNPs identified from transcriptome sequencing of six cultivated potato cultivars using either Sanger or Illumina transcriptome sequencing [18] and genotyping carried out using an Illumina iScan Reader utilizing the Infinium HD Assay Ultra by Gen-Probe, Manchester.

DNA samples were arranged in two 96-well plates, each in an arrangement of 12 columns by 8 rows. The parents Stirling and 12601ab1 were in rows 1 and 2 of column 1 on plate 1, followed by the offspring. The intensities of the fluorescent dyes associated with the two alleles of the SNP are expressed as Cartesian coordinates (X,Y) by the programme Genome Studio. After normalisation, Genome Studio transforms the intensities to a combined SNP intensity R = (X+Y) and an intensity ratio theta = (2/π)*arctan(Y/X) [22] i.e. to polar coordinates. The theta score gives information about the dosages of each allele for the parents and offspring and was exported from Genome Studio for further analysis.

### Data pre-processing

Three criteria were used to identify SNP theta scores as suitable for further analysis: a trimmed range greater than 0.1, no strong spatial trend and no missing values.

Range: An ideal SNP for which all possible dosages (AAAA, AAAB, AABB, ABBB, BBBB) can be observed is expected to consist of theta scores in five clusters, centred around 0.0, 0.25, 0.5, 0.75 and 1.0 respectively. This has a range of 1.0. The lowest expected range is for a simplex SNP, for example with parents AAAA x AAAB, where parents and offspring will have scores clustered around 0.0 and 0.25, and so a range of 0.25. Inspection of the data suggested the use of a trimmed range, between the 2% and 98% quantiles, to remove the influence of a small number of outliers. SNPs were retained in the set for analysis if the trimmed range was greater than or equal to 0.1.

Spatial trend: Index plots of the theta scores against the order of the samples on the plates showed that for some SNPs there was a spatial trend and/or a difference between the plates. To address this, the theta score was modelled as a smooth function of the sample order using locally-weighted regression (loess) with four degrees of freedom [23]. The spatial trend was most apparent when the range was small, with 77% of SNPs with a trimmed range less than 0.1 showing a trend that was significant with p<0.0001. However excluding the SNPs with a small range as described above did not remove all the SNPs showing a spatial trend. Therefore if the smooth trend was significant with p<0.0001, the SNP was excluded. This threshold was chosen by inspection of the theta scores to establish how severe spatial trend needed to be to affect a visual classification.

Missing values: There were a small number of SNPs with missing theta scores and for computational ease these were excluded.

### Allele dosage estimation

#### Possible SNP configurations

Table 1 shows the possible genotype configurations and the probabilities for each dosage at a SNP for the parents and offspring in a full-sib autotetraploid mapping population. This assumes random chromosomal segregation i.e. no preferential pairing, no double reduction and no segregation distortion. The table lists 13 configurations, of which all but the first segregate in the offspring. In the table, configurations have been arranged so that in each case the expected theta value for parent 1 is less than or equal to that of parent 2: further configurations are obtained by permuting the parents. The abbreviations S, D, SS etc in Table 1 will be used to refer to different configurations; simplex or duplex will also be described as NxS, NxD etc when it is important to distinguish which parent is heterozygous.

**Table 1:** Possible parent and offspring genotype configurations in an autotetraploid species.

Parent 1	Parent 2	Type	P1 theta	P2 theta	Dosage probabilities				
					AAAA0	AAAB0.25	AABB0.5	ABBB0.75	BBBB1.0
1 group									
AAAA	BBBB	Null (N)	0	1	0	0	1	0	0
2 groups									
AAAA	AAAB	Simplex (S)	0	0.25	1/2	1/2	0	0	0
ABBB	BBBB	Simplex (S)	0.75	1	0	0	0	1/2	1/2
AAAA	ABBB	Triplex (T)	0	0.75	0	1/2	1/2	0	0
AAAB	BBBB	Triplex (T)	0.25	1.0	0	0	1/2	1/2	0
3 groups									
AAAA	AABB	Duplex (D)	0	0.5	1/6	4/6	1/6	0	0
AABB	BBBB	Duplex (D)	0.5	1	0	0	1/6	4/6	1/6
AAAB	AAAB	Double- simplex (SS)	0.25	0.25	1/4	2/4	1/4	0	0
ABBB	ABBB	Double-simplex (SS)	0.75	0.75	0	0	1/4	2/4	1/4
AAAB	ABBB	X-double-simplex (XSS)	0.25	0.75	0	1/4	2/4	1/4	0
4 groups									
AAAB	AABB	Simplex- duplex (SD)	0.25	0.5	1/12	5/12	5/12	1/12	0
AABB	ABBB	Duplex- simplex (DS)	0.5	0.75	0	1/12	5/12	5/12	1/12
5 groups									
AABB	AABB	Double-duplex (DD)	0.5	0.5	1/36	8/36	18/36	8/36	1/36

Possible genotype configurations at the parents and the associated dosage probabilities for their F1 offspring. P1 theta and P2 theta show the expected theta scores for parents 1 and 2.

**Figure 1:**
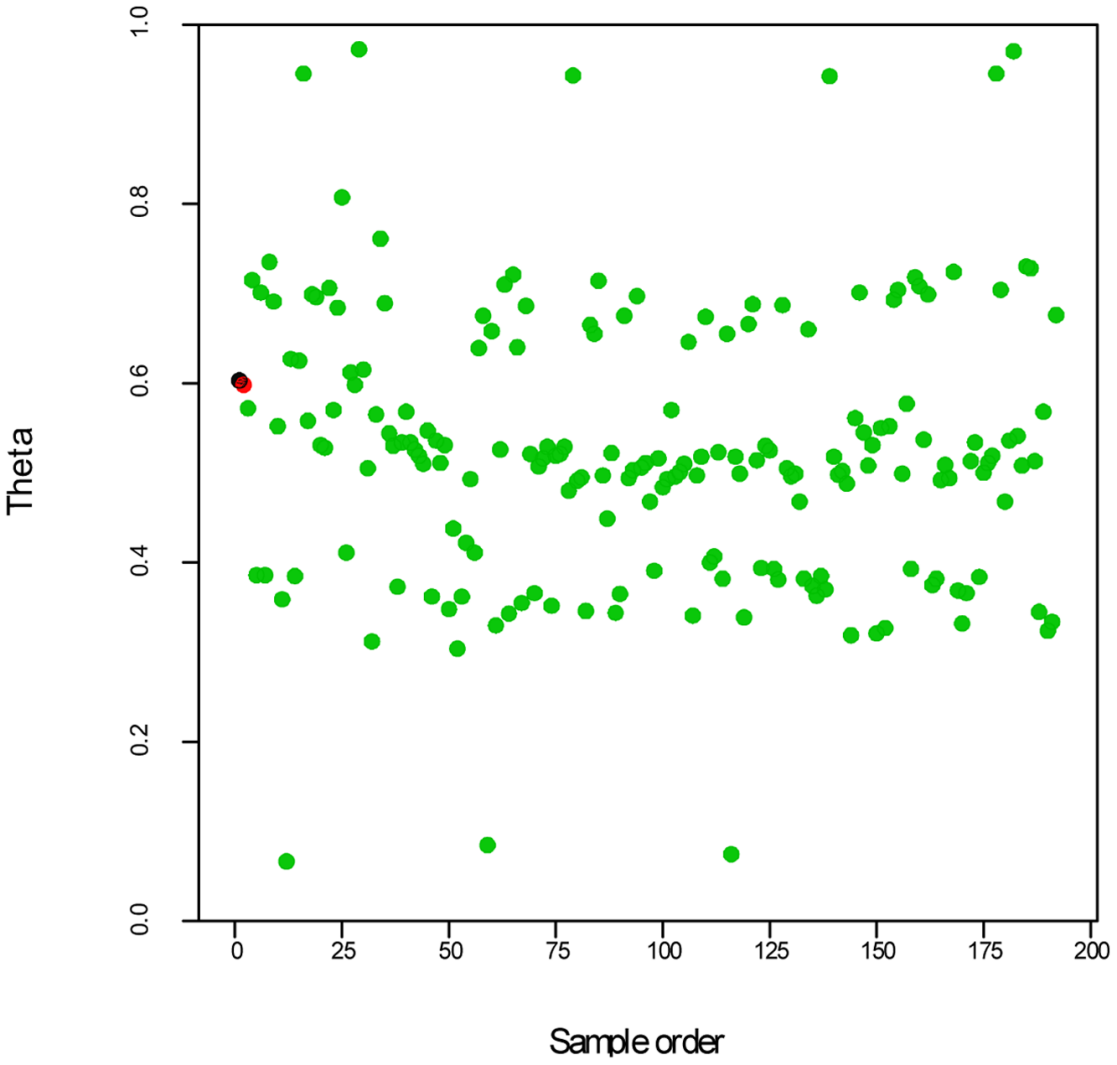
An example of theta scores for a SNP with five dosage classes. A plot of theta scores against the sample order for SNP c1_10069, with Stirling in black and 12601ab1 in red.

Figure 1 shows an order plot for solcap_snp_c1_10069. This plausibly has five dosage classes visible, and both parents (red and black crosses) are in the central category as would be expected for a double-duplex ‘five category’ SNP. The challenge is to develop an approach to test whether the SNP data is a good fit to the expected category probabilities, and to compare the fit for the different configurations.

#### Normal mixture model approach

A normal mixture model can be used to model the theta scores of the offspring. This will have from one to five component distributions, depending on how many dosage categories are possible for that configuration. The mixture proportions will be considered as given, using the information in Table 1. As discussed by [11], the mixture means can be unevenly spaced if the relationship between the intensity and the allele dosage differs between the A and B alleles. The mixture means are therefore estimated for each SNP, together with a constant variance for all dosages.

Let the theta scores be *y_P1_* and *y_P2_* for the parents and *y_1_…y_n_* for the *n* offspring. Let A be the more frequent allele, and B the less frequent. Let the mixture proportions for configuration *C* be *p_C_(g)*, where *g* = 0…4 corresponding to the possible dosages, expressed as the number of ‘B’ alleles. The likelihood of the offspring data under configuration *C* can be written as
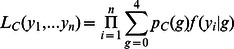
where 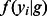, the probability density function of the theta score conditional on the dosage g, is a normal distribution with mean *μ_g_* and variance *σ^2^*.

The following derivation is adapted from [24], which demonstrated how the EM algorithm can be used to reduce fitting the mixture model to two steps, fitting a weighted regression, and updating the mixture weights.

The maximum likelihood equations are
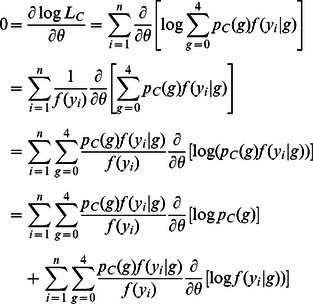
where 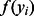 is the (unconditional) probability density function of the theta scores and 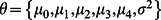.


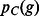
 does not depend on *θ*, so the first term is zero. The second term represents a regression of the offspring theta scores as a function of the dosage, and weighted by the probability of each dosage given the theta score 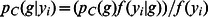.

#### Implementation of the EM algorithm

For configuration *C* with *k* dosage classes, the algorithm consists of the following steps:

1. A *k*-mean cluster analysis [25]. This uses as initial values the expected theta values for configuration *C*. For example, if configuration *C* is an AAAA x AAAB model, the initial cluster means are 0.0 and 0.25. The final cluster means are used for the next step.
2. Initial expectation step. Calculate the probability of each observed theta value arising from each dosage class 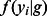 using the cluster means and an overall estimate of the initial variance. From this, calculate the weighted probability 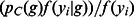.
3. Initial maximisation step. Carry out a weighted regression of the theta values on the dosages to obtain updated estimates of the dosage class means and variance.
4. Repeat steps 2 and 3 until the log-likelihood converges.
5. Allocate each offspring to a dosage class based on the maximum posterior probability 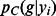.

The full likelihood for the parents and offspring is then computed as
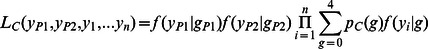
where 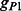 and 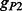 are the dosage classes of the parents for configuration *C*. The minimum Bayesian information criterion (BIC) [26] is used to compare all configurations *C*, including the null configuration, to determine which is the most likely. This is defined as
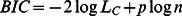
where *p* is the number of estimated parameters, equal to the number of class means plus one (for the variance).

A chi-square goodness-of-fit test is used to test the segregation of each SNP by comparing the numbers of offspring in each dosage class of the selected configuration with the theoretical proportions in Table 1.

### Linkage mapping of the simplex SNPs and preliminary identification of homologous chromosomes

While it is possible to cluster all the non-distorted SNPs together and then carry out a global linkage analysis of each linkage group, a preliminary step of ordering the simplex SNPs and making tentative associations of these into chromosomal groups for each parent is a useful check. This makes the later analyses, especially the overall cluster analysis, easier to interpret. Simplex SNPs can be scored as 1 or 0, depending on whether they carry the simplex allele or not. Recombination fractions between pairs of simplex SNPs are calculated in the same way as other simplex markers, using the equations given by [19],[27]. The equation for the recombination fraction and LOD score between simplex SNPs in coupling phase is the same as for several marker types in diploid populations and so software for diploid populations can be used. The simplex SNPs were analysed using JoinMap 4.0 [28] for each parent separately, with the SNPs coded as a phase-known doubled-haploid type population. This gives the correct calculation of recombination fraction and LOD scores for simplex SNPs in coupling, but does not estimate any repulsion linkages. The SNPs were grouped on the basis of a recombination fraction of at most 0.25 to another member of that group. For a population of this size, the grouping is equivalent to separating the SNPs using a LOD of 10.8. Within the groups the SNPs were ordered using the regression mapping method implemented in Joinmap 4.0, which estimates distances between SNPs using the weighted least squares procedure of [29], and checked for any indications of poor fit.

Work on dominant markers [19] has shown that duplex+simplex pairs of markers are the most informative for identifying linkages among homologous chromosomes, while simplex+double-simplex markers in coupling phase are informative for identifying associations between the parental groups [15]. These papers give the expected phenotype frequencies for pairs of dominant simplex, duplex and double-simplex markers. For SNP data, consider a simplex SNP AAAA x AAAB linked to a duplex SNP CCCC x CCDD, or to a double-simplex SNP CCCD x CCCD, with recombination fraction *r*. In each case six genotype classes are found in the offspring. Table 2 gives the phenotypic frequencies for different phases of these pairs for SNP data. Initial associations can be established using chi-square tests of independent segregation of pairs of SNPs, as discussed by [13]. These tests were carried out between all simplex SNPs and all duplex SNPs from the same parent, and between simplex and double-simplex SNPs. In the latter case, only significant simplex+double-simplex coupling pairs were used to associate parental groups, as the simplex+double-simplex repulsion pairs have much lower power to detect non-independent segregation.

**Table 2:** Expected genotype class frequencies for a simplex SNP linked to a duplex or double-simplex SNPs in coupling or repulsion phase in an autotetraploid species.

Offspring class	Simplex genotype (dosage)	Linked SNP genotype (dosage)	Simplex+duplex coupling	Simplex+duplex repulsion	Simplex+double-simplex coupling	Simplex+double-simplex repulsion
1	AAAA (0)	CCCC(0)	(1−r)/6	r/6	(1−r)/4	(1+r)/12
2	AAAA (0)	CCCD(1)	2/6	2/6	1/4	3/12
3	AAAA (0)	CCDD(2)	r/6	(1−r)/6	r/4	(2−r)/12
4	AAAB (1)	CCCC(0)	r/6	(1−r)/6	r/4	(2−r)/12
5	AAAB (1)	CCCD(1)	2/6	2/6	1/4	3/12
6	AAAB (1)	CCDD(2)	(1−r)/6	r/6	(1−r)/4	(1+r)/12

### Clustering the SNPs

SNPs with significant distortion were excluded before clustering into linkage groups. For simplex SNPs, the threshold for exclusion was if the significance of the chi-square goodness-of-fit statistic was less than 0.001. Our experience with analysing other types of markers in tetraploid populations is that distortion in other marker configurations can give more problems in map assembly, and so for duplex and higher dosages the threshold applied was to exclude the SNP if the significance of the chi-square goodness-of-fit statistic was less than 0.01. The SNPs were clustered into linkage groups using a chi-square test for independent segregation as described by [13]. As the chi-square statistics are not directly comparable between SNP pairs with different numbers of dosage classes (i.e. different degrees of freedom), the significance level of the test was used as a measure of distance between a pair of SNPs. Group average cluster analysis was used, and a range of thresholds were explored to investigate how the SNPs should be partitioned. Two cluster analyses were run: the first consisted of the simplex and duplex SNPs from Stirling together with double-simplex and higher dosage SNPs, and the second consisted of the simplex and duplex SNPs from 12601ab1 together with double-simplex and higher dosage SNPs. Clusters from the two analyses were combined manually, excluding any higher dosage SNPs where the allocation was only supported by one of the analyses.

### Estimation of the recombination fractions and LOD scores between pairs of SNPs

When pairs of SNPs are being considered, with different configurations and different phases, there are a very large number of possibilities. The expected class dosage frequencies could be derived for each configuration, as done for simplex+duplex pairs etc in Table 2, and maximum likelihood equations for the recombination fractions can be derived from these, although these generally cannot be solved analytically. Luo et al. [13] developed a general computer algorithm to handle all possible configurations for dominant and codominant markers, and this can be extended to analyse SNPs with dosage information. Here we review this algorithm briefly, and apply it to two linked duplex SNPs as an example.

Let S and T be two linked SNPs with recombination fraction *r* between them and write the parental genotype as S_a_T_a_/S_b_T_b_/S_c_T_c_/S_d_T_d_ to represent the alleles on the four homologous chromosomes. At gamete formation, we assume that all three possible pairings into bivalents are equally likely, with probability 1/3: (S_a_T_a_/S_b_T_b_ and S_c_T_c_/S_d_T_d_, S_a_T_a_/S_c_T_c_ and S_b_T_b_/S_d_T_d_ or S_a_T_a_/S_d_T_d_ and S_b_T_b_/S_c_T_c_). Within each bivalent, there are three types of gamete: non-recombinant (four genotypes), single recombinant (eight genotypes) or double recombinant (four genotypes), with probabilities (1−*r*)^2^/4, *r*(1−*r*)/4 or *r* ^2^/4. For the first bivalent pairing above, examples would be S_a_T_a_S_c_T_c_, S_a_T_a_S_c_T_d_ and S_a_T_b_S_c_T_d_ respectively. A general equation for the frequency of a gametic genotype can be written as
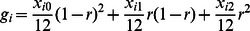
where *x_i0_*, *x_i1_* and *x_i2_* are the numbers of nonrecombinants, single recombinants and double recombinants for genotype *i*.

As an example, consider a parent with two duplex SNPs linked in coupling. Let SNP S have alleles A and B, and SNP T have alleles C and D, and let the dosage measure the number of B and D alleles respectively. The parental genotype is AC/AC/BD/BD. One possible bivalent pairing gives the homozygous bivalents AC/AC and BD/BD and the gamete ABCD is obtained regardless of the degree of recombination i.e. the counts *x_i0_*, *x_i1_* and *x_i2_* are 4,8,4 for this gamete. The other two bivalent pairings gives heterozygous bivalents AC/BD and AC/BD, with nine possible gametes. The coefficients *x_i0_*, *x_i1_* and *x_i2_* are summarised in Table 3.

**Table 3:** Gametic genotype frequencies for two duplex SNPs (AAAA x AABB and CCCC x CCDD) linked in coupling.

Gametic genotype	Dosage of allele B	Dosage of allele D	*x_i0_,*	*x_i1_*	*x_i2_*
AACC	0	0	2	0	0
AACD	0	1	0	4	0
AADD	0	2	0	0	2
ABCC	1	0	0	4	0
ABCD (from AC/AC and BD/BD pairing)	1	1	4	8	4
ABCD (from AC/BD and AC/BD pairing)	1	1	4	0	4
ABDD	1	2	0	4	0
BBCC	2	0	0	0	2
BBCD	2	1	0	4	0
BBDD	2	2	2	0	0

*x_i0_*, *x_i1_* and *x_i2_* are the numbers of nonrecombinants, single recombinants and double recombinants for each genotype.

A random union of the gametes from the two parents gives zygotes derived from zero to four recombinations, and a general equation for the zygote genotype *i* can be written as
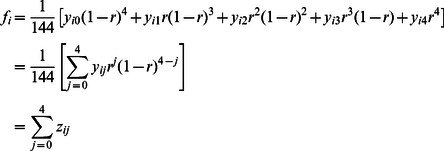
The computer algorithm developed by [13] to calculate the coefficients {*y_ij_*} for dominant or codominant markers was modified to give the coefficients associated with each dosage class.

If *n_i_* offspring have genotype *i*, for genotype classes *i* = 1…*k*, the log-likelihood for the recombination fraction *r* can be written as
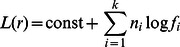
and the maximum likelihood equation for *r* can be obtained by setting the derivative of the log-likelihood with respect to *r* equal to zero. Luo et al. [13] show how the EM algorithm can be used to obtain an iterative solution for the maximum likelihood equation. The log-likelihood is calculated for each possible phase of the pair of SNPs. The most likely phase is taken as the one with the highest maximum of the log-likelihood, and 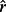 is the corresponding estimate from that phase. The LOD score is evaluated as the difference between the log-likelihood (using base 10) at *r* = 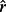 and that at *r* = 0.5 for the most likely phase.

Luo et al. [13] showed that different configurations of dominant and codominant markers give different amounts of information about linkage. To quantify this for SNP markers with dosage information, a small simulation study was carried out. Tetraploid offspring were simulated under a model of random chromosomal segregation, from parents with genotypes containing all possible SNP pairings as adjacent markers, linked in each case with an expected recombination fraction of 0.1. Two hundred offspring were simulated for each of 20 populations. Recombination fractions and LOD scores between adjacent markers were estimated as described above.

### Ordering the SNPs

The above analysis was used to estimate the most likely phase, the recombination fraction and the LOD score for all pairs of SNPs within each of the twelve linkage groups. This information can be used to order the SNPs and estimate the distances between them, for example using the weighted least squares procedure of [29] implemented in the regression mapping method of JoinMap 4.0. As there were very large numbers of SNPs in each cluster, any SNP that was a near-duplicate of another (differing for at most two of the 190 offspring) was excluded from the ordering. Two rounds of the JoinMap algorithm were used to estimate a map of SNPs that fit well according to JoinMap's chi-square criterion. Any remaining SNPs were given a more tentative allocation adjacent to the mapped SNP with which they had the highest LOD score, provided that the estimate of the recombination fraction was less than 0.05. Other SNPs remained unplaced on the map.

The final step in the map construction was to reconstruct the phase of the complete linkage group. The simplex SNPs provide a framework for the linkage group, ideally identifying all four homologous chromosomes from each parent, and other SNPs were placed relative to these. A computer routine was written to check the most likely phase of each non-simplex SNP with respect to each simplex marker, and alleles were designated as in coupling or repulsion with each if the LOD score was greater than 5.0. Any SNP with an unknown or incomplete phase after this step had its phase assigned manually, using SNP pairs with as high a LOD as possible and also a high LOD difference between the most likely and the second most likely phase.

### Map checking by QTL mapping of the theta scores

The linkage map can be checked by QTL interval mapping of the original theta scores on the linkage map. This gives a check of the position of the SNP, and whether the phase has been identified correctly. For example, if a SNP has been identified as a simplex SNP on chromosome d of parent 1, we expect the theta score for that SNP to map to the SNP position, and for the coefficient associated with chromosome d of parent 1 to be the only significant coefficient.

The approach used for QTL interval mapping is similar to that proposed by [14], with modification of the estimation of the QTL genotype probabilities to take into account the dosage information. This is followed by fitting a QTL mixture model to model the theta scores as a function of the QTL genotypes.

#### Estimation of QTL genotype probabilities

In order to carry out a QTL analysis, we need to estimate QTL genotypes in the offspring from the parental genotypes and phases, and the offspring dosages. There are 36 possible genotypes (assuming no double reduction). Previous work [14] with dominant and codominant markers considered, for each offspring, which of the 36 possible genotypes give the observed marker data at each position. For dosage data, we can similarly consider which genotypes give the observed dosages. Let the chromosomes be numbered a–d for parent 1, and e–h for parent 2. Table 4 gives a small example for a single offspring.

**Table 4:** An example of QTL genotype estimation for one offspring from the parental genotypes and the offspring dosage.

SNP	Parental genotypes								Offspring dosage	No. possible genotypes
	a	b	c	d	e	f	g	h		
M1	A	A	B	B	A	A	B	B	4	1 (cdgh)
M2	A	A	A	A	A	B	A	A	1	18
M3	A	A	A	B	B	B	A	A	2	15
M4	A	A	B	B	A	B	A	B	4	1 (cdfh)

Parent 1 has chromosomes labelled a–d and parent 2 has chromosomes e–h. ‘No. possible genotypes’ shows the number of offspring genotypes giving the observed dosage. See text for further explanation.

In this example, only one genotype is possible for each of SNPs M1 and M4, cdgh for M1 and cdfh for M4. For M2 there are 18 possible genotypes (i.e. every configuration including chromosome f). For M3, there are 15 possible genotypes that give the observed dosage. We infer that there must have been a recombination between M1 and M2, as M1 has chromosomes g and h from parent 2 and M2 has chromosome f. This is compatible with a bivalent pairing of f with g and e with h in parent 2. The genotype cdfh will give the observed dosages for SNPs M2 and M3 as well as M4. Therefore this sequence of dosages can be obtained from one recombination in parent 2, between SNPs M1 and M2, and no recombination in parent 1 over this section. All other possible reconstructions would involve more recombination events.

In general, for a chromosome with *K* SNPs, we obtain a 36 by *K* matrix for each offspring indicating whether the observed offspring dosage can be obtained from that genotype or not.

Hackett et al. [14] used a branch and bound algorithm to search for sequences of genotypes and recombination positions that give the observed sequence, with as few crossovers as possible. Usually there are several possible sequences, so we obtain the probabilities of the offspring having each possible genotype at each SNP position. However the branch and bound method had the disadvantage of not allowing for any errors in the scoring process. It is also too slow to be extended to high density maps. Therefore we have used an alternative approach to this step, based on a hidden Markov model (HMM) [30].

A HMM has a series of states, forming a path. Transition probabilities model the transitions from one state to another. From each state, symbols are emitted, modelled by emission probabilities. Only the symbols are observable, and the aim is to infer the path of states from the observed symbols.

Denote the path of states as {π_i_, *i* = 1…*K*} and the sequence of observed symbols as {*x_i_*, *i* = 1…*K*}. The transition probability between states is given by
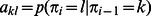
and is independent of the states at earlier times. The emission probability is given by
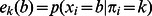
and depends on the state of the hidden variable at time *i*.

To apply this to a single offspring, consider the 36 genotypes to be the unobservable states at SNP positions 1 to *K* along the chromosome, where positions here replaced the usual HMM ‘times’. Associated with each genotype is an indicator *s_ik_*, equal to 1 if the offspring dosage at position *i* can be obtained from genotype *k*, and 0 otherwise. The observed symbols are a sequence of *K* symbols ‘y’, indicating correct matches between the genotypes and the offspring dosages. The set of emitted symbols from each state is {y,n}. If the SNP data is regarded as error-free, the emission probabilities are
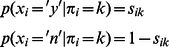
However the SNP data may have been scored incorrectly, with probability λ. The emission probabilities therefore become
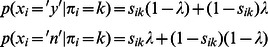
The transition probabilities depend on the distance between SNPs, and the number of recombinations to move between two states. At this point it is necessary to take into account the process of bivalent formation. Under the model of random chromosomal segregation, the chromosomes pair at meiosis into bivalents, and then recombination occurs within each bivalent. (In the small example above the observed dosages could not occur if chromosomes c and d have paired together in parent 1, or if chromosomes g and h have paired together in parent 2.) We model this by considering each of the bivalent pairings separately, and then combining the results, weighted by the overall probability of observing the symbol sequence from that bivalent.

For example, consider bivalent pairing chromosome a with b and c with d in parent 1 and e with f and g with h in parent 2.

If the state at position *i* is aceg, then if there are no recombinations the process will remain in state aceg at position *i*+1. To move to state bceg, adeg, acfg or aceh requires 1 recombination. Other possible moves and the number of recombinations are shown in Table 5. For this bivalent, some states cannot occur e.g. abef.

**Table 5:** Number of recombinations required to reach possible genotype states from starting genotype state of aceg.

		Chromosomes from parent 2			
		eg	eh	fg	fh
Chromosomes from parent 1	ac	0	1	1	2
	ad	1	2	2	3
	bc	1	2	2	3
	bd	2	3	3	4

This is based on a bivalent pairing of chromosome a with b and c with d in parent 1 and e with f and g with h in parent 2.

The transition probabilities are therefore
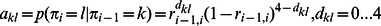
where *r* is the recombination fraction between positions *i*−1 and *i*, and *d_kl_* is the number of recombinations between those states. A preliminary analysis used an approximate value of 0.01 for each recombination fraction, as there are approximately 100 SNPs mapped by JoinMap on each chromosome, with a length of approximately 100 cM. This is a regular HMM. The model was then extended to a position-dependent HMM, using the estimated recombination fractions for adjacent SNPs, and this is presented here.

From the HMM, we need to estimate the probabilities under each bivalent model *B* of each of the different genotype states along the path,
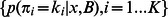
These are known as the posterior state probabilities and can be solved by use of recursive algorithms, the forward algorithm and the backward algorithm [31]. The scaling method for these algorithms, as described there, was used to avoid computing underflow errors. These give the posterior state probabilities of each genotype at each position, along with the overall probabilities of the sequence *p(x|B)*, each conditional on a particular bivalent pairing. The overall genotype probabilities at each map position are then calculated as
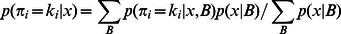
This gives QTL genotype probabilities at the SNP positions along the chromosome. To map QTLs along the entire chromosome requires QTL genotypes to be estimated between the SNP positions by interpolation. A cubic spline [32] was used to interpolate the probabilities with a 1 cM spacing.

#### Model for a quantitative trait

There are various models with different levels of complexity that can be used for quantitative traits. Kempthorne [33] expressed the expected value of a quantitative trait in a tetraploid individual A_i_A_j_A_k_A_l_ as the sum of a population mean, the main effects of the alleles and diallelic, triallelic and tetraallelic interactions. For our full sib family model, with parent 1 having chromosomes a–d and parent 2 having chromosomes e–h, an individual will inherit alleles A_i_ and A_j_ from parent 1 with *i,j* taking values from (ab, ac, ad, bc, bd, cd). Likewise it will inherit A_k_ and A_l_ from parent 2, with *k,l* taking values from (ef, eg, eh, fg, fh, gh). Let *X_i_*, *i* = a…h be 0/1 indicator variables corresponding to allele A_i_ being absent/present for that individual. As each individual inherits 2 alleles from each parent, we have the constraint that *X_a_*+*X_b_*+*X_c_*+*X_d_* = 2 and *X_e_*+*X_f_*+*X_g_*+*X_h_* = 2, and corresponding constraints on interactions among [12]. A full model could fit parameters to all of the 36 possible genotype classes, but this would be prone to over-fitting to the data.

In modelling the theta scores for each SNP, we expect them to be a simple function of the number of B alleles at the corresponding SNP, and therefore we model them using the main effects of each allele in Kempthorne's model, assuming interaction terms to be zero. Let the main effects of alleles A_a_…A_h_ be *α_a_….α_h_*. A main effects model, taking into account the constraints on *X_i_*, has the form
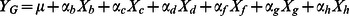
This model is fitted by an iterative weighted regression on the QTL genotype probabilities, as described by [14]. The number of iterative steps was limited to ten to reduce the possibility of over-fitting to the data.

A small simulation study was used to test how well the QTL genotypes were reconstructed by the HMM. Using the maps for each of the 12 chromosomes, 100 individuals were simulated so that their genotypes at each SNP were known. The HMM was used to estimate the genotype probabilities, and this was compared with the known genotypes.

The theta scores for the SNPs used by JoinMap were then analysed, mapping each set of theta scores on the expected chromosome.

## Results

### Data pre-processing

For 6176 of the 8300 SNPs, the trimmed range for this population was greater than or equal to 0.1. Exclusion of SNPs showing a significant trend (with p<0.0001) reduced the set further to 5609 SNPs. There were only 23 SNPs among the remaining set with missing values. While most had fewer than ten missing values, for computational ease these were excluded. This gave a final dataset of 5586 SNPs.

### Allele dosage estimation

Normal mixture models were fitted to the theta scores for each of the 5586 SNPs obtained after pre-processing. For most SNPs one model was clearly indicated. Unevenly spaced mixture means were detected, but did not complicate the genotype designations. In general the posterior probabilities for allocating offspring to genotype classes were high, with a mean posterior probability of 0.982, and a mean number of 180.0 of the 190 offspring having a posterior probability greater than or equal to 0.90. Table 6 shows how these SNPs were assigned to the configurations, using the minimum BIC. The null configuration corresponds to a single normal distribution having the minimum BIC. A chi-square test of goodness of fit was used to compare the counts of each dosage class with the expected values in Table 1. The last column of Table 6 shows the numbers of SNPs that were distorted at a significance level *p*<0.001. The proportion of distorted SNPs is higher for configurations with larger numbers of classes, suggesting that these may be harder to fit. A total of 5332 SNPs were classified as segregating, rather than the null configuration, and 520 of these were distorted with *p*<0.001. The theta scores for these 520 distorted SNPs were checked graphically and the BIC scores re-examined to see if these suggested that an alternative configuration had a similar BIC. This showed 46 SNPs that resembled simplex SNPs graphically (i.e. two similar sized groups), but where a small number of outliers or some trend led to the EM algorithm fitting more than two groups. These 46 were recoded as simplex SNPs to give a total of 5378 SNPs for linkage analysis.

**Table 6:** Distribution of SNPs into genotype classes.

Type	Stirling	12601ab1	Count	p<0.001
Null			254	n/a
Simplex from Stirling	AAAB	AAAA	926	23
Simplex from 12601ab1	AAAA	AAAB	788	22
Triplex from Stirling	ABBB	AAAA	119	4
Triplex from 12601ab1	AAAA	ABBB	103	6
Duplex from Stirling	AABB	BBBB	487	66
Duplex from 12601ab1	AAAA	AABB	392	57
Double-simplex	AAAB	AAAB	635	41
X-double-simplex	AAAB	ABBB	188	54
Simplex-duplex	AAAB	AABB	644	94
Duplex-simplex	AABB	ABBB	629	92
Double-duplex	AABB	AABB	421	61

The final column shows the number of SNPs that are distorted from the expected class frequencies with p<0.001 according to a chi-square test of goodness of fit. n/a = not applicable.

### Linkage mapping of the simplex SNPs

After exclusion of the SNPs that were distorted with *p*<0.001, and the addition of the recoded simplex SNPs, there were 1040 simplex SNPs from Stirling and 887 from 12601ab1. The SNPs from Stirling segregated into 51 groups of 4–65 SNPs, while the SNPs from 12601ab1 segregated into 45 groups of 4–75 SNPs. In general the groups were easily ordered, with few SNPs having problems with poor fit or many double recombinants. The recoded simplex SNPs were found to fit well on the maps.

Duplex-simplex SNP pairs with significant non-independent segregations (*p*<0.001) were used to tentatively identify homologous chromosomes. For Stirling, eight groups with four homologous chromosomes, three groups with three homologous chromosomes, one group with five homologous chromosomes (two of which were very short) and one isolated group were identified. For 12601ab1, six groups with four homologous chromosomes, four with three chromosomes, two with five chromosomes (again, two were very short) and one isolated chromosome were identified, giving twelve groups and one isolated chromosome for each parent. Significant simplex to double-simplex coupling pairs identified matches between the chromosomes from each parent, giving 12 tentative linkage groups.

The simplex SNPs were also combined with simplex AFLPs and simplex SSR alleles scored on this population to compare the linkage groups with those of [1] (data not shown). This showed that seven tentative groups of SNPs corresponded to chromosomes I (Ia and Ib combined), II, III, IV, V, VI and VIII of [1]. The other five groups of SNPs corresponded to groupings XIa, XIb and C combined, A and F combined, B and D. N. Subramanian (unpublished PhD thesis) has constructed an AFLP and DArT map of this population and has compared this with the diploid potato map of [34] to identify D, A/F, XIa, XIb/C, and B as chromosomes VII, IX, X, XI and XII respectively.

### Clustering the SNPs

After elimination of the distorted SNPs, 3205 SNPs were used for the first cluster analysis, with 1405 simplex and duplex SNPs from Stirling and 1800 double-simplex and higher dosage SNPs. The second cluster analysis had 2995 SNPs, 1195 simplex and duplex SNPs from 12601ab and the 1800 double-simplex and higher dosage SNPs. An inspection of different clustering thresholds suggested a choice of *p* = 0.1 be made for the average significance for partitioning. At this level, there were 30 clusters from the analysis of the Stirling and higher dosage SNPs, and 32 groups from the analysis of the 12601ab1 and higher dosage SNPs. The clusters typically consisted of simplex SNPs from groups identified above as homologous together with higher dosage makers, but longer simplex coupling groups were regularly split up. These were merged manually if they consisted of simplex SNPs mapped together by the JoinMap analysis. Corresponding clusters from the two analyses were merged manually, ensuring that all higher dosage SNPs were clustered in their groups by the analyses from both parents, rather than just one. Twelve clusters of SNPs resulted, and comparison with [18] confirmed the earlier identification of the clusters as chromosomes I–XII. Table 7 shows the numbers of SNPs on each chromosomal group.

**Table 7:** The number of SNPs on each chromosome, the number of near-duplicates and the number mapped after two rounds of JoinMap analysis.

Chromosome	No. SNPs from cluster analysis	No. near-duplicates	No. omitted	No. mapped by JM	No. placed in bins
I	491	182	45	142	122
II	406	173	14	120	99
III	362	154	49	74	85
IV	496	172	54	152	118
V	396	158	54	119	65
VI	447	186	41	122	98
VII	213	59	13	89	52
VIII	301	81	33	85	102
IX	364	103	105	91	65
X	290	88	33	104	65
XI	309	75	75	85	74
XII	300	109	22	118	51
Total	4375	1540	536	1301	998

A near-duplicate differs for at most 2/190 offspring from another SNP.

### Simulation study of the estimation of the recombination fractions and LOD scores

**Figure 2:**
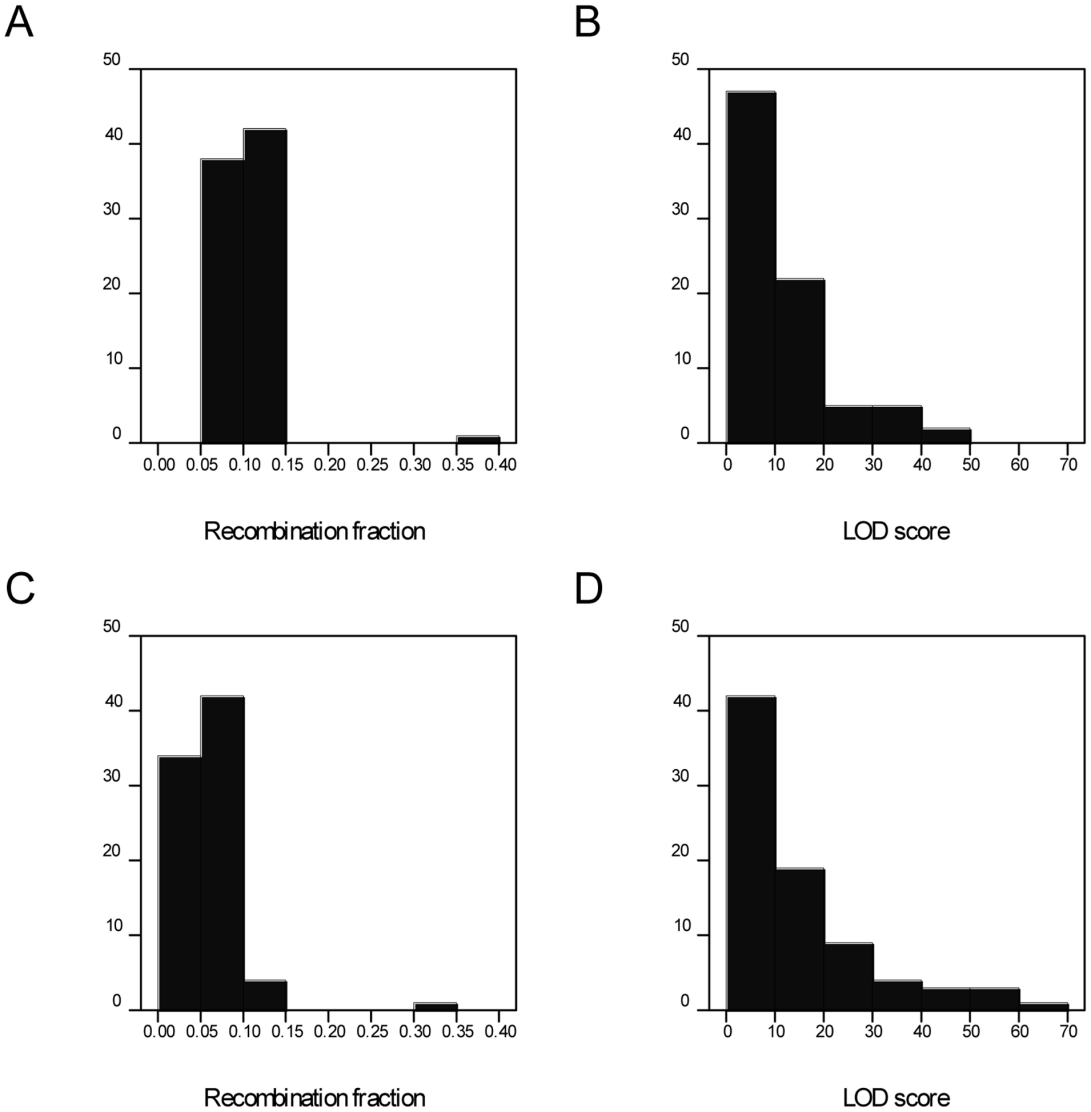
Distribution of statistics estimated from simulated SNP pairs. (A) Histogram of the estimated recombination fractions, simulated with expectation 0.1. (B) Histogram of the corresponding estimated LOD scores. (C) Histogram of the estimated recombination fractions, simulated with expectation 0.05. (D) Histogram of the corresponding estimated LOD scores.

In Table 1, the simplex and triplex configurations can be treated as a single class for linkage analysis. Considering all possible pairs of SNPs in all phases, there are a total of 67 pairs to be investigated. Simulations were carried out for pairs with true recombination fractions of 0.1 and 0.05. For brevity in the results below, we present the figures for the true recombination fraction of 0.1 first, followed by that for the true recombination fraction of 0.05 in brackets. The mean recombination fractions and mean LOD scores were calculated over 20 populations of 200 offspring for each value of the recombination fraction. Figure 2 shows histograms of the mean recombination fractions and the corresponding LOD scores. Most of the mean recombination fractions are close to the simulated values of 0.1 (0.05), with one outlier in each case with mean recombination fraction of 0.38 (0.34). These were from the same configuration, consisting of a XSS SNP linked to a SS SNP, in repulsion in each parent. This configuration also had the lowest mean LOD score, of 0.58 (0.69).

Traditionally a LOD of 3.0 is used to infer linkage, but most LOD scores here are much higher than this. For dominant markers, simplex+simplex (S+S) markers in coupling phase have the highest LOD scores. For this study, these had a mean LOD score of 32.1 (41.3). Four configurations had higher mean LOD scores: XSS+XSS in coupling phase (mean LOD 43.1 (62.1)), SS+SS in coupling phase (mean LOD 40.5 (57.2)), DS+DS in coupling phase (mean LOD 38.9 (59.7)) and DD+DD in coupling phase (mean LOD 37.8 (58.6)). For the SS+SS configuration in coupling phase, this LOD can be compared with the expected LOD of 22.9 (32.6) for this population if the marker was scored as a dominant marker without dosage information (using the formula in [15]). Likewise the mean LOD score for a pair of duplex SNPs linked in coupling was 26.6 (37.5), compared with an expected LOD of 14.8 (21.1) if these were scored as dominant markers.

The worst configuration for dominant markers is S+S in repulsion phase, which here had mean LOD scores of 3.5 (3.8). Sixteen SNP configurations had lower mean LOD scores than this. Most of these were configurations containing simplex SNPs in repulsion phase or duplex SNPs in mixed phase, for example a DD SNP linked to a NxD SNP in mixed phase (mean LOD 0.9 (1.1)), or a DS SNP linked to a DS SNP in mixed phase in the first parent and repulsion in the second parent (mean LOD 1.6 (2.6)). Other configurations with low mean LOD scores were a DS SNP linked to a SD SNP in coupling in one parent and repulsion in the other (mean LOD 2.3 (3.1)), and a DD SNP linked to a SS SNP in coupling in one phase and repulsion in the other (mean LOD 1.6 (1.8)). If a pair of SNPs with one of these less informative configurations were tested in isolation, they would be wrongly classed as unlinked. However in a larger set of SNPs with a range of configurations, linkage can be established via a third SNP in a more informative configuration.

The configurations were also examined to see whether the correct phase was always inferred. For some SNP pairs, two phases are possible, but give the same recombination fraction and LOD score. An example is a pair of DD SNPs, linked in coupling in one parent and in mixed phase in the other, where the CxM and MxC phases cannot be distinguished unless information from additional SNPs is included. Apart from such pairs, the phase was inferred correctly for the configurations with high LOD scores. For the configuration DS linked to DS in CxR phase, with mean LOD 8.5 (11.6), the phase was inferred wrongly as MxC for one of the 20 simulated populations with recombination fraction 0.1 (but was always inferred correctly when the recombination fraction was 0.05). However the recombination fractions and LOD scores were very similar. For the configuration SD linked to SS in CxR phase, with mean LOD 5.7 (8.6), the phase was inferred wrongly as RxC for one of the 20 simulated populations with recombination fraction 0.1, and for XSS linked to DS in CxC phase, mean LOD 5.5 (9.8), two of the 20 populations with recombination fraction 0.1 were wrongly inferred as RxR. For the simulations with true recombination fraction 0.05, the configuration SS linked to SD in RxC phase, with mean LOD 2.4 (3.7), the phase was inferred incorrectly for three of the simulated sets, twice as CxC and once as CxR. These were the only incorrect inferences of phase for configurations with a mean LOD score greater than 3, but incorrect inferences became more common for configurations with mean LOD score below 3.

On the basis of this small study, we conclude that this approach identifies the correct phase and estimates recombination fractions precisely for all but the least informative configurations. Using dosage information leads to higher LOD scores than when the corresponding configuration is scored as presence/absence. For the least informative configurations, some linkages will be missed, and some phases may be inferred incorrectly, and the probability of this happening increases with increased marker separation, as for presence/absence markers. However incorrect inferences can be avoided by using information from additional markers in more informative configurations. We found no cases of an incorrect inference with a high LOD score.

### Ordering the markers

After removal of the near-duplicate SNPs, recombination fractions and LOD scores were calculated between all pairs of SNPs in each chromosomal group for each possible phase, and the recombination fractions and LOD scores with the highest log-likelihood were written to a pairwise data file for analysis with JoinMap 4. The following JoinMap options were used: the Haldane mapping function; using only pairs of SNPs with recombination fractions less than 0.45 and LOD scores greater than 0.05, and to run a ripple search after every five SNPs were added to the map. Two mapping rounds were run with JoinMap, and the JoinMap diagnostics to check the fit of the mapped SNPs were examined.

It was found that XSS SNPs gave particular problems in the map assembly. As there were only a small number of these, it was decided to omit this class of SNPs from the JoinMap analysis. Isolated simplex SNPs placed into a chromosomal group by the cluster analysis but unlinked to any of the other simplex SNPs were also omitted from the JoinMap analysis. The map obtained after two rounds of JoinMap was used as the first estimate of the linkage map (the ‘mapped’ SNPs). Other SNPs were placed in approximate bins according to their maximum LOD with the mapped SNPs, provided the recombination fraction was less than 0.05. These are referred to as the binned SNPs.

**Figure 3:**
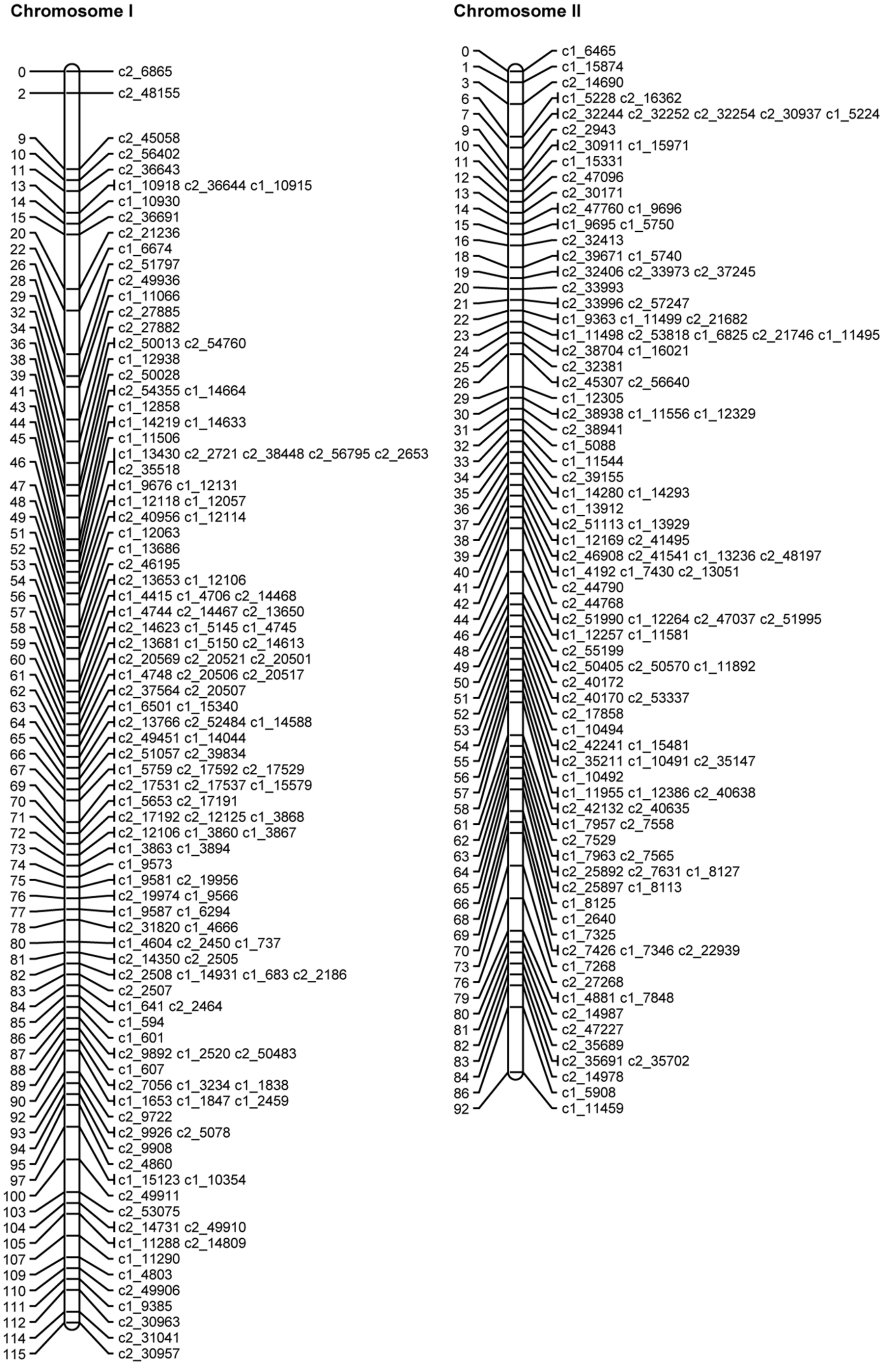
Linkage maps for potato chromosomes I and II.

**Figure 4:**
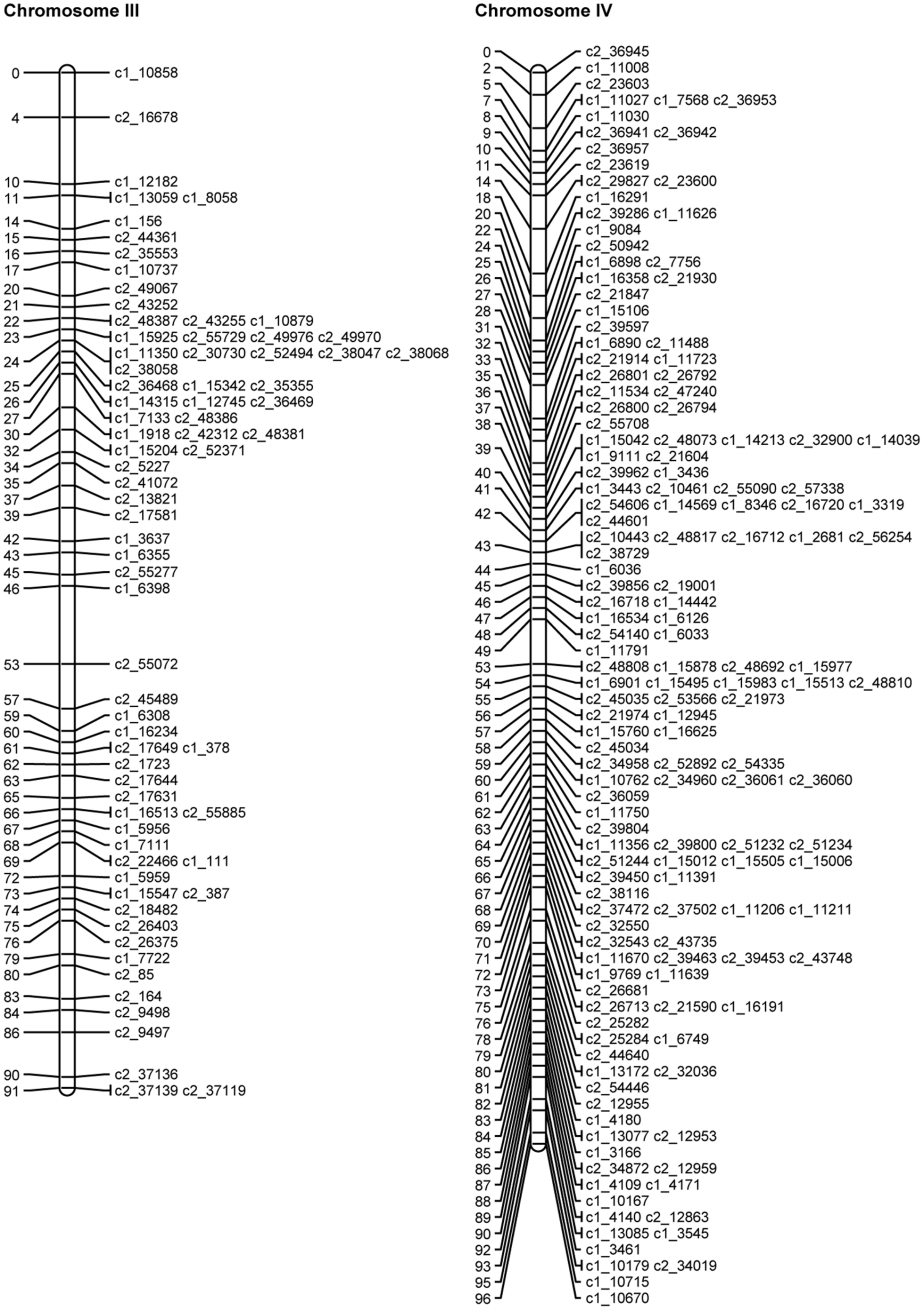
Linkage maps for potato chromosomes III and IV.

**Figure 5:**
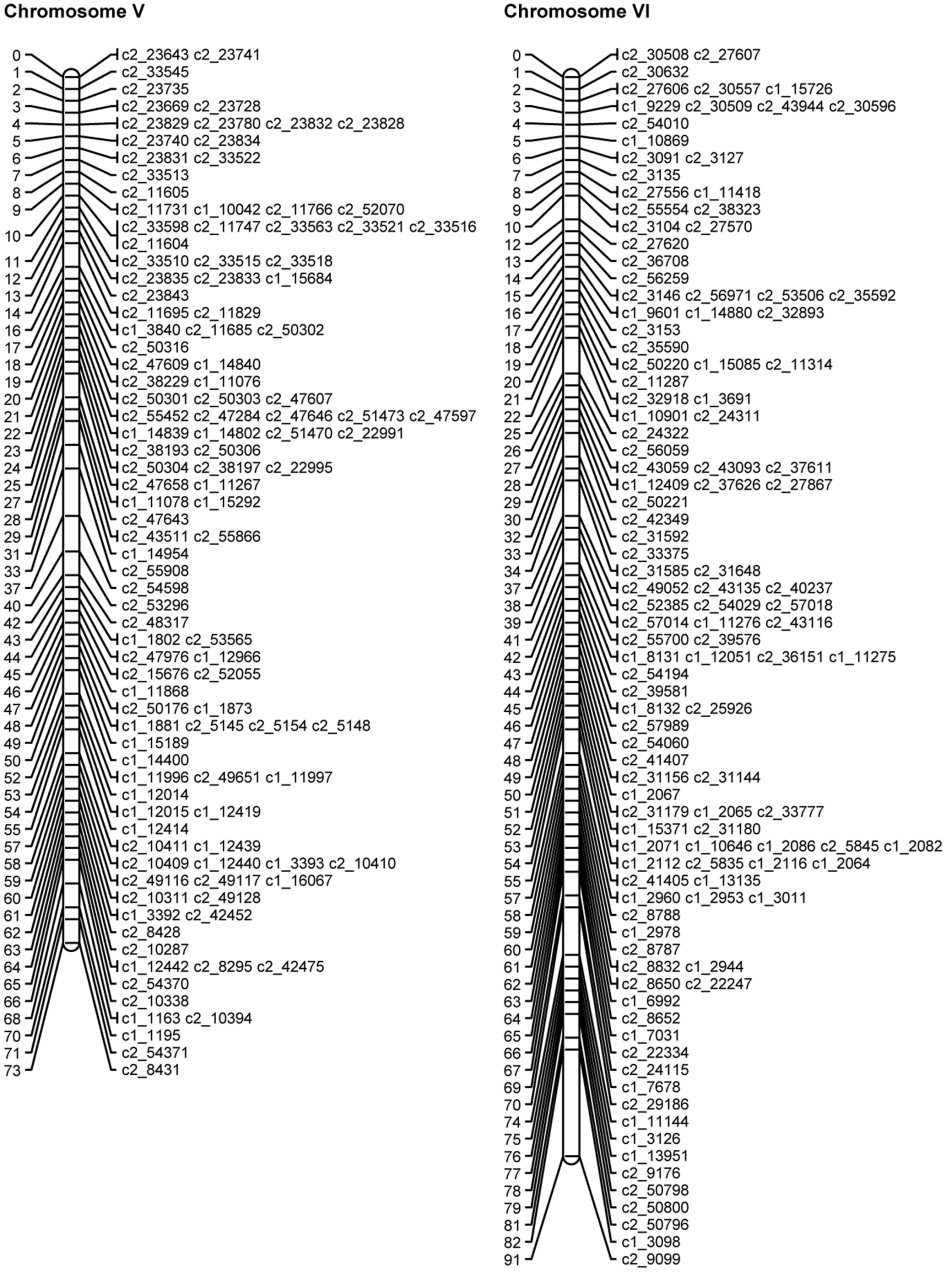
Linkage maps for potato chromosomes V and VI.

**Figure 6:**
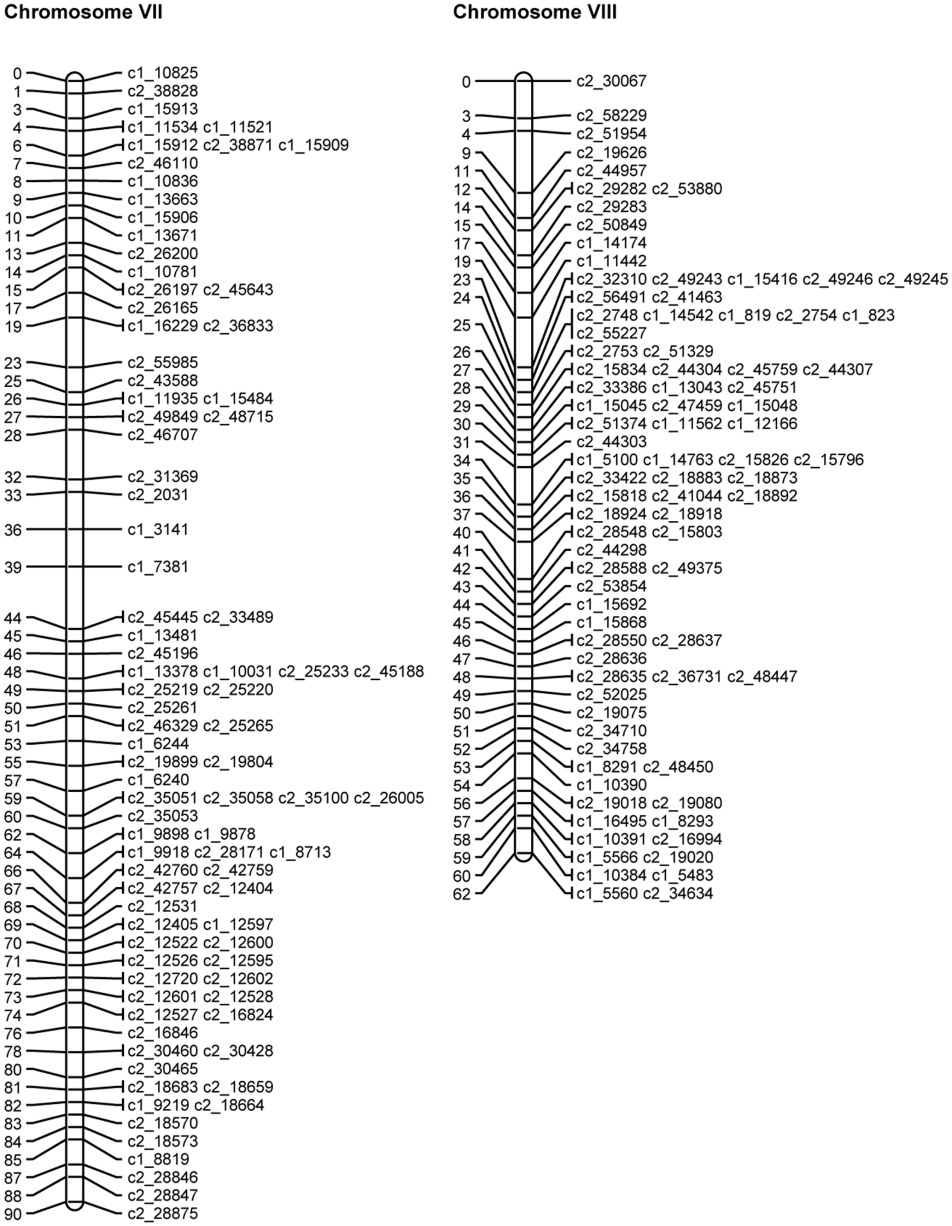
Linkage maps for potato chromosomes VII and VIII.

**Figure 7:**
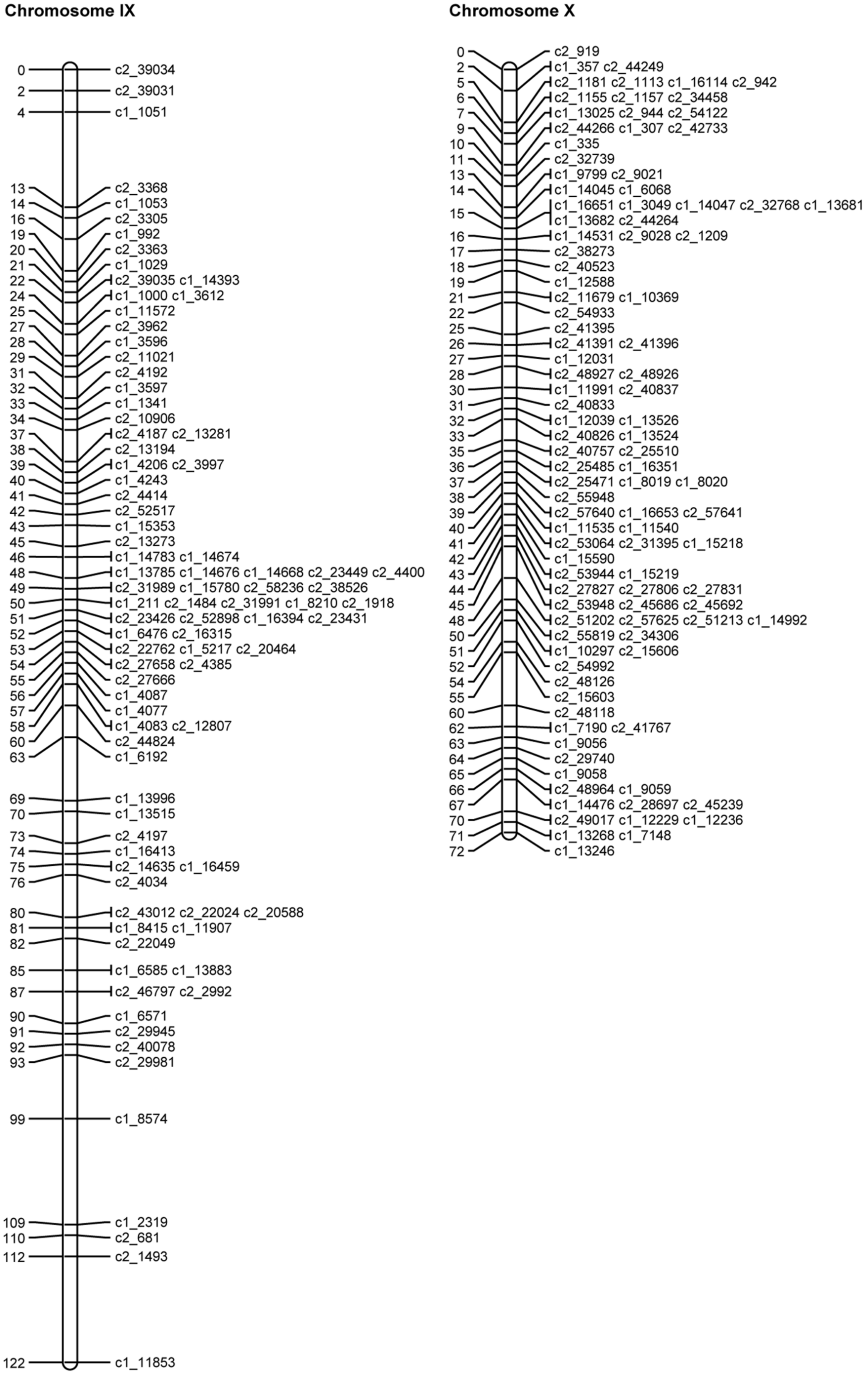
Linkage maps for potato chromosomes IX and X.

**Figure 8:**
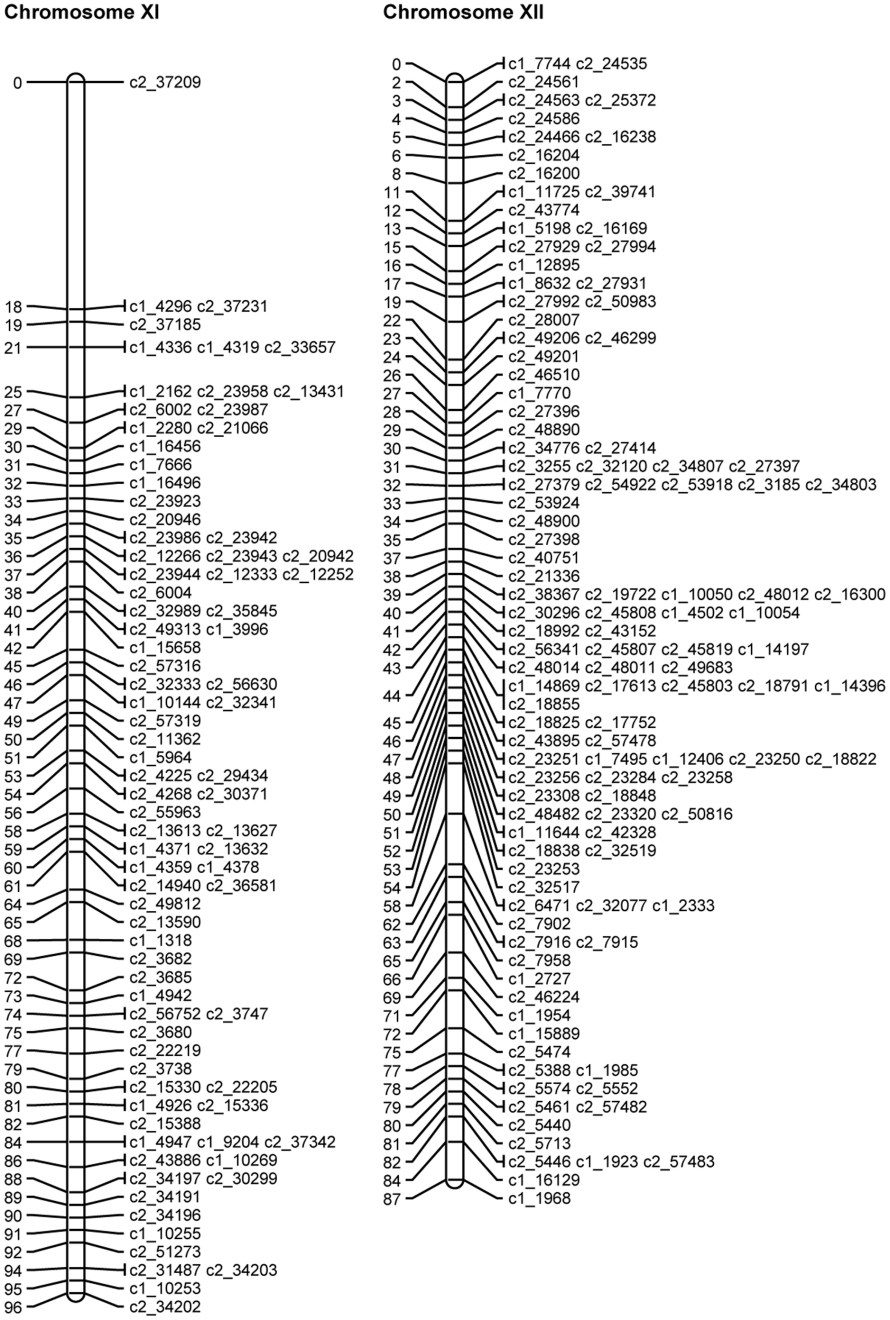
Linkage maps for potato chromosomes XI and XII.

SNP phases were estimated for the mapped and binned SNPs as described above. The QTL mapping approach (see details below) was then used to map the theta scores for the mapped and binned SNPs as quantitative traits on the linkage map of mapped SNPs. Where discrepancies were identified, the SNPs in question were removed and the JoinMap analysis and QTL analysis were rerun. Table 7 gives the final numbers of omitted SNPs, mapped SNPs and binned SNPs for each chromosome. The length, calculated using the mapped SNPs, varied from 71.9 cM to 121.6 cM, with a total length of 1087.5 cM. Figures 3, 4, 5, 6, 7, 8 show the linkage maps of the mapped SNPs, and Tables S1, S2, S3, S4, S5, S6, S7, S8, S9, S10, S11, S12 gives details of the mapped, binned and duplicate SNPs for each chromosome. The posterior probabilities for allocating offspring to dosage classes using their theta scores were checked for each type of SNP: the mean posterior probabilities are 0.996, 0.983 and 0.956 for the mapped and duplicate SNPs, the binned SNPs and the SNPs omitted due to distorted segregation or unclear theta scores, and the corresponding mean numbers of offspring (out of 190) with posterior probabilities greater than or equal to 0.90 are 187.6, 180.0 and 165.8 respectively.

### QTL mapping as a check on the linkage map

A small simulation study was used to check how well the HMM reconstructed individuals with known genotypes, simulated from the final SNP maps. One hundred offspring were simulated for each chromosome. Table 8 summarises the result as the proportion of genotypes where the correct four chromosomes were identified (out of 100*K*, where *K* is the number of SNPs on the chromosome). It also summarises the proportion of genotypes where three chromosomes out of four were correctly identified. The proportion reconstructed correctly was greater than 0.75 for nine of the twelve chromosomes. The three chromosomes with lower proportions reconstructed correctly had the highest proportions of simplex SNPs, which are only informative about the presence/absence of a single chromosome. They also were among the least dense of the maps. Although it was not investigated here, it would be possible to include additional higher dosage SNPs from the binned set to improve the coverage for these chromosomes.

**Table 8:** Summary statistics for each chromosomal group, and the proportion of simulated genotypes where the correct chromosomes were identified.

Chromosome	No mapped by JM	Length (cM)	Ratio No/Length	No simplex (%)	Proportion correct (3/4 correct)
I	142	115.3	1.23	43 (0.303)	0.815 (0.143)
II	120	91.9	1.31	27 (0.225)	0.835 (0.104)
III	74	91.5	0.81	29 (0.392)	0.751 (0.140)
IV	152	95.8	1.59	46 (0.303)	0.821 (0.120)
V	119	73.1	1.63	34 (0.286)	0.841 (0.106)
VI	122	90.8	1.34	32 (0.262)	0.767 (0.174)
VII	89	90.0	0.99	47 (0.528)	0.571 (0.136)
VIII	85	62.3	1.36	22 (0.259)	0.762 (0.171)
IX	91	121.6	0.75	37 (0.407)	0.717 (0.132)
X	104	71.9	1.45	26 (0.250)	0.853 (0.083)
XI	85	96.2	0.88	35 (0.412)	0.647 (0.152)
XII	118	87.1	1.35	32 (0.271)	0.877 (0.081)

QTL mapping was then used to check the position, the %variance explained and the inferred phase of the mapped and binned SNPs when their theta scores were mapped as quantitative traits. We will look at chromosome V in detail. This had 396 SNPs according to the cluster analysis, of which 158 were removed as near-duplicates, giving 238 for the JoinMap analysis. Eleven XSS SNPs and eight isolated simplex SNPs were also excluded before the JoinMap ordering was run. The first analysis mapped 99 SNPs, and left 120 unmapped. Six of the unmapped SNPs were too far from any of the mapped SNPs to be placed in bins, but the remaining 114 were allocated to bins. All the mapped SNPs, binned SNPs and the 19 SNPs excluded from the JoinMap run were analysed by QTL mapping of the theta scores.

For the mapped SNPs, the %variance explained ranged from 85.6% to 99.4%, with a mean of 94.0%. One SNP mapped as a QTL to a position 11 cM from its location as a marker, but the other 98 mapped SNPs had less than 5 cM between the locations as markers and as QTLs, with mean displacement 0.8 cM. For the binned markers, the %variance explained ranged from 77.0% to 99.4%, with a mean of 92.3%. There were greater displacements between the locations as markers and as QTLs, with a mean displacement of 3.9 cM and a maximum displacement of 31 cM. Two of the SNPs excluded from the JoinMap analysis had lower %variance explained, at 65% and 68% respectively. For comparison, 100 random traits generated from a standard normal distribution were analysed by the same approach to look at the distribution of the %variance: this had a mean of 4.0% and an upper 95% point of 9.4%.

All SNPs with %variance <85% or a discrepancy between the SNP phase and the significant QTL coefficients were inspected graphically. A further 33 SNPs (7 mapped, 26 binned) were excluded as a result of this process, due to trend or excess scatter leading to uncertainty in the SNP genotype inference. The JoinMap ordering was rerun without these SNPs, giving a final map of 119 mapped SNPs, 65 binned SNPs and two unmapped SNPs that were too far from the mapped SNPs to be placed in a bin. The QTL mapping analysis was rerun and no further problems were found. The mean displacement between positions as markers and positions as QTLs of the remaining binned markers decreased to 3.0 cM, with a maximum displacement of 14 cM. This is still higher than for the mapped markers, probably due to the lower precision of the position of the binned markers. Table 9 illustrates the phase information and QTL coefficients for three of the mapped markers.

**Table 9:** QTL coefficients for three examples of SNPs mapped on LG V.

SNP	Map pos. (cM)	Phase	QTL pos. (cM)	μ	α_b_	α_c_	α_d_	α_f_	α_g_	α_h_	Av. se	R^2^
c2_22991	23.2	AABA AABA	24	−0.021	0.037	0.233	0.009	0.014	0.248	0.035	0.006	95.8%
c1_15189	49.9	ABAB AAAA	50	0.047	0.143	0.004	0.147	0.006	0.003	−0.003	0.005	88.1%
c2_49117	59.5	AAAB AABB	59	0.075	0.006	−0.002	0.202	0.021	0.248	0.241	0.009	91.6%

Av. Sed shows the average standard error of the chromosome coefficients α_b_–α_h_. R^2^ gives the percentage variance of the theta score explained by the QTL.

### Double reduction

**Figure 9:**
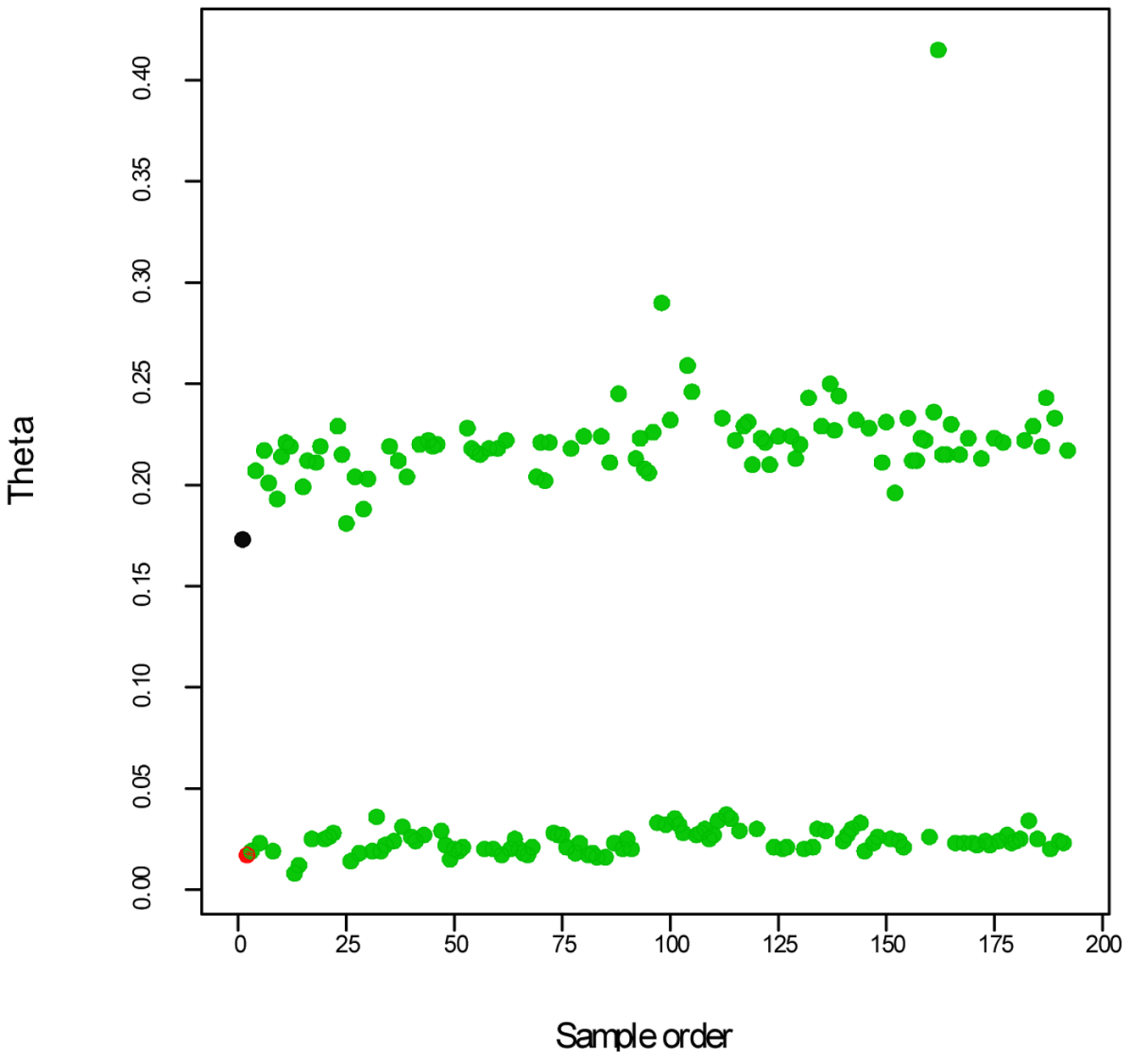
An example of theta scores showing a double reduction product. A plot of the theta scores for simplex SNP c2_45058 at 8.6 cM on LG I shows a probable double reduction product for individual 160. This individual showed a similar pattern for the linked SNP c2_6865 at 0.0 cM.

This analysis has assumed a model of random chromosomal segregation, with bivalents forming and recombination within these. However segregation in autotetraploids is more complicated, as reviewed by [35],[35]. Double reduction results in two sister chromatids occurring in a single gamete. This occurs when a multivalent is formed with a cross-over between a locus and the centromere, and the two pairs of chromatids migrate to the same pole at the first meiotic division. Double reduction may lead to some offspring having dosages that are not possible under the model of random chromosomal segregation. This is most obvious for the configurations having two categories of offspring under random chromosomal segregation, simplex and triplex SNPs. For a simplex SNP, AAAA x AAAB, double reduction can produce AABB offspring. For a triplex SNP, AAAA x ABBB, double reduction can produce AAAA offspring. The simplex and triplex SNP data was re-examined for double reduction products. To do this, the theta scores were regressed on the dosage class to estimate the difference *d* between the theta scores for the two dosage classes. A double reduction product is expected to be separate from these classes i.e. to have a large residual. Any SNP where there were residuals greater than 0.75*d were inspected graphically and the identities of individuals with double reduction products noted. An example is given in Figure 9, which shows the theta scores for simplex SNP c2_45058 at 8.6 cM on LG I with a probable double reduction product for individual 160. This individual showed a similar pattern for the linked SNP c2_6865 at 0.0 cM.

This analysis was complicated by trend in the theta scores for some SNPs. It was not always clear whether extreme values were due to double reduction products or to trend. Double reduction was therefore inferred only for individuals with a double reduction product for two or more SNPs linked in coupling on the same homologous chromosome but at separate locations. For Stirling, double reduction products were detected for all but one chromosomal group. The maximum number of individuals where double reduction has occurred on a chromosomal group (i.e. on any of the 4 homologous chromosomes) was six of the population of 190, with a mean number of 2.7 individuals. For 12601ab1, double reduction products were detected for eight of the twelve chromosomal groups. The maximum number of individuals was 13, with a mean of 3.4 individuals. We conclude that the rate of double reduction in this population is low, but could be usefully explored more once issues of trend have been addressed.

## Discussion

In this paper we have developed statistical methodology to construct SNP linkage maps in autotetraploid species, making use of the additional information given by the SNP allele dosages, in contrast to current methods based on allele presence/absence data. This permits the use of SNP configurations that contain little or no information with presence/absence data. For example, a triplex SNP AAAA x ABBB will result in offspring that all carry both the A and B alleles (unless double reduction occurs), but dosage information will separate the AAAB offspring from the AABB, so that this SNP can be mapped. Similarly, for a simplex by duplex SNP AAAB x AABB, all offspring will carry the A allele and the ratio presence to absence of B will be 11∶1 (in the absence of double reduction), which is too high to be informative, while the dosage data identifies four classes with 0–3 B alleles. Another advantage is that the precision of the estimates of recombination fraction may be increased by the use of dosage information compared to presence/absence information: we have demonstrated this for the case of duplex and double-simplex SNPs. The methods developed here are suitable for linkage mapping of any genotypic data derived from a technology that is informative about allele dosages for tetraploid species. We anticipate that these methods can be further extended to look at dosages derived from read counts obtained from genotyping by sequencing [10].

The methods have been applied to construct a linkage map in the well-studied potato cross between cultivar Stirling and breeding line 12601ab1, using 190 progeny. The analysis was complicated by the discovery of some spatial trend related to sample order in the allele intensity ratios (theta scores) derived from the SNP technology employed (Illumina iSelect) as well as the Genome Studio software. This was most apparent in the non-segregating SNPs with a low range for the theta scores, but could also be found in some segregating SNPs. SNPs with a strong trend were excluded from the analysis, but some further SNPs with trends were detected and removed later, when theta values were mapped as quantitative traits. All of this analysis has been carried out using with summary-level data from Genome Studio, i.e. one observation per probe type, per sample genotype. Ritchie et al. [37] and Dunning et al. [38] have discussed the pre-processing of this type of data and have both suggested that working with image-level or bead-level data can improve the assays by identifying and removing sources of spatial trend. There is scope for further research here. There is also scope for further analysis of the scores from Genome Studio, including the R scores as well as the theta scores, and considering whether more markers can be added to the map if null alleles are taken into account. We would recommend that the assay could be made more robust against spatial trend by an improved design, with the parents of the mapping population on every plate analysed and in the centre of the sample plate rather than at the end. For some SNP configurations, accurate parental information is essential, most notably distinguishing between the simplex by duplex and duplex by simplex configurations, and replicated parents would help this.

Methodology has also been tested for QTL mapping, using a hidden Markov model to infer QTL genotype probabilities in the offspring and modelling the traits as a function of these. So far these methods have only been applied to theta scores, where it is expected that very high proportions of the variance should be explained by a single location on the linkage map, and that the effects of the separate alleles on the traits are likely to be additive. Further testing is in progress to establish suitable thresholds for using this method for regular phenotypic traits, controlled by several QTLs and with a higher proportion of environmental variation, and to compare different models for the traits, including interactions between alleles.

The map constructed here contains 3839 mapped markers, either mapped directly or in ‘bins’. Previous maps constructed for this population contained far fewer markers (<500), which were primarily dominant AFLPs, with a smaller number of co-dominant SSRs [1]–[15],[17]. Other, predominantly diploid, potato linkage maps published to date also contain a much smaller number of markers, for example the studies of [39] which analysed 303 and 419 segregating AFLP markers in the two populations studied and [40], who used 384 segregating AFLP and SSR loci. A notable exception is the Ultra High Density (UHD) genetic map of potato [41] which contained more than 10,000 AFLP markers, which were placed into segregation ‘bins’ due to the computational issues associated with such a large data set, and the relatively small size of the population (136 individuals). Perhaps equally as important as the increased marker number is the sequence based nature of the predominantly genic SNPs deployed here. The SNP panel was selected so as to target single copy regions of the genome, so for the majority of them it is possible to assign them a unique genomic location in the published potato genome [18]. This direct ‘map to genome’ link has major implications for those aiming to identify candidate genes at trait loci, whereby the set of potato genes flanked by any set of SNP markers can now be identified in a facile manner. Work is in progress to compare and integrate the map reported here, along with one of a diploid cross made using the same SNP panel, to information on the potato genome sequence [34]. Supplementary Figure S1 shows a preliminary comparison between the SNPs on chromosome I of this cross and the order of the SNPs that are in common with chromosome I of the diploid DRH cross from [18], as shown in their Figure 1. These types of analyses can lead to the identification of potential errors in genome assembly and in reconstruction of chromosomal ‘pseudomolecules’ and to insights about the genetic and physical properties of the potato genome, and will ultimately lead to further improvements in the genome sequence. This study further demonstrates the utility of the potato 8300 SNP array, first reported by [18].

These data can be made available to other researchers: please contact the authors for details.

## Supporting Information

Figure S1 **Comparison of the tetraploid linkage map for chromosome I with that estimated on diploid potato population DRH by Felcher et al.** [**18**] **.**
(TIF)

Table S1 **Order of the mapped, binned and duplicate SNPs on chromsomes I–XII.** The (near) duplicate and binned markers are listed below the closest mapped marker. The recombination fraction of each binned marker with the closest mapped marker is shown in the final column.
(XLS)

Table S2 **Order of the mapped, binned and duplicate SNPs on chromsomes I–XII.** The (near) duplicate and binned markers are listed below the closest mapped marker. The recombination fraction of each binned marker with the closest mapped marker is shown in the final column.
(XLS)

Table S3 **Order of the mapped, binned and duplicate SNPs on chromsomes I–XII.** The (near) duplicate and binned markers are listed below the closest mapped marker. The recombination fraction of each binned marker with the closest mapped marker is shown in the final column.
(XLS)

Table S4 **Order of the mapped, binned and duplicate SNPs on chromsomes I–XII.** The (near) duplicate and binned markers are listed below the closest mapped marker. The recombination fraction of each binned marker with the closest mapped marker is shown in the final column.
(XLS)

Table S5 **Order of the mapped, binned and duplicate SNPs on chromsomes I–XII.** The (near) duplicate and binned markers are listed below the closest mapped marker. The recombination fraction of each binned marker with the closest mapped marker is shown in the final column.
(XLSX)

Table S6 **Order of the mapped, binned and duplicate SNPs on chromsomes I–XII.** The (near) duplicate and binned markers are listed below the closest mapped marker. The recombination fraction of each binned marker with the closest mapped marker is shown in the final column.
(XLS)

Table S7 **Order of the mapped, binned and duplicate SNPs on chromsomes I–XII.** The (near) duplicate and binned markers are listed below the closest mapped marker. The recombination fraction of each binned marker with the closest mapped marker is shown in the final column.
(XLS)

Table S8 **Order of the mapped, binned and duplicate SNPs on chromsomes I–XII.** The (near) duplicate and binned markers are listed below the closest mapped marker. The recombination fraction of each binned marker with the closest mapped marker is shown in the final column.
(XLS)

Table S9 **Order of the mapped, binned and duplicate SNPs on chromsomes I–XII.** The (near) duplicate and binned markers are listed below the closest mapped marker. The recombination fraction of each binned marker with the closest mapped marker is shown in the final column.
(XLS)

Table S10 **Order of the mapped, binned and duplicate SNPs on chromsomes I–XII.** The (near) duplicate and binned markers are listed below the closest mapped marker. The recombination fraction of each binned marker with the closest mapped marker is shown in the final column.
(XLS)

Table S11 **Order of the mapped, binned and duplicate SNPs on chromsomes I–XII.** The (near) duplicate and binned markers are listed below the closest mapped marker. The recombination fraction of each binned marker with the closest mapped marker is shown in the final column.
(XLS)

Table S12 **Order of the mapped, binned and duplicate SNPs on chromsomes I–XII.** The (near) duplicate and binned markers are listed below the closest mapped marker. The recombination fraction of each binned marker with the closest mapped marker is shown in the final column.
(XLS)
