# Supplementary figures and images for: Linkage Analysis and QTL Mapping Using SNP Dosage Data in a Tetraploid Potato Mapping Population
Source: PLoS One. 2013 May 21;8(5):e63939. doi: 10.1371/journal.pone.0063939 (PMC3660524; doi:10.1371/journal.pone.0063939)

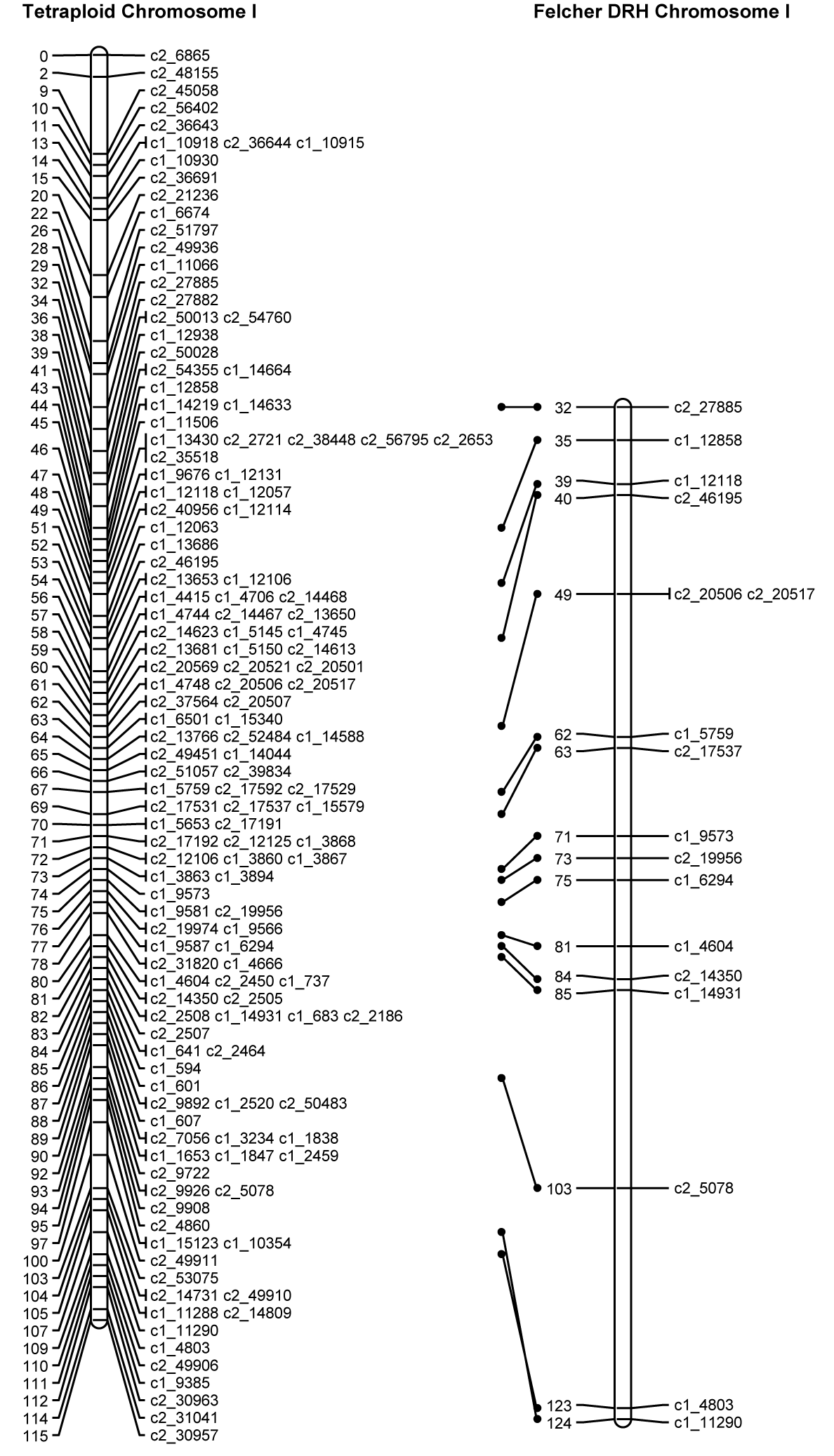

Supplement: Figure S1 — Comparison of the tetraploid linkage map for chromosome I with that estimated on diploid potato population DRH by Felcher et al. [18] . (TIF) [file pone.0063939.s001.tif]
